# The NOAA NCEI marine microplastics database

**DOI:** 10.1038/s41597-023-02632-y

**Published:** 2023-10-20

**Authors:** Ebenezer S. Nyadjro, Jennifer A. B. Webster, Tim P. Boyer, Just Cebrian, Leonard Collazo, Gunnar Kaltenberger, Kirsten Larsen, Yee H. Lau, Paul Mickle, Tiffany Toft, Zhankun Wang

**Affiliations:** 1 Northern Gulf Institute, Mississippi State University, 1021 Balch Blvd, Stennis Space Center, MS USA; 2 NOAA National Centers for Environmental Information, 1021 Balch Blvd, Stennis Space Center, MS USA; 3 NOAA National Centers for Environmental Information, Silver Spring, MD USA; 4 General Dynamics Information Technology, Stennis Space Center, MS USA; 5 Present Address: Vesta, PBC, San Francisco, CA USA

**Keywords:** Ocean sciences, Environmental sciences

## Abstract

Microplastics (<5 mm) pollution is a growing problem affecting coastal communities, marine ecosystems, aquatic life, and human health. The widespread occurrence of marine microplastics, and the need to curb its threats, require expansive, and continuous monitoring. While microplastic research has increased in recent years and generated significant volumes of data, there is a lack of a robust, open access, and long-term aggregation of this data. The National Oceanic and Atmospheric Administration (NOAA) National Centers for Environmental Information (NCEI) now provides a global open access to marine microplastics data on an easily discoverable and accessible GIS web map and data portal (https://www.ncei.noaa.gov/products/microplastics). The objective of this data portal is to develop a repository where microplastics data are aggregated, archived, and served in a user friendly, consistent, and reliable manner. This work contributes to NCEI’s efforts towards data standardization, integration, harmonization, and interoperability among national and international collaborators for monitoring global marine microplastics. This paper describes the NOAA NCEI global marine microplastics database, its creation, quality control procedures, and future directions.

## Introduction

Microplastics are defined as plastics that are smaller than 5 mm (0.20 in) and are a growing problem affecting coastal communities, marine ecosystems, marine life, and human health^1–5^. Microplastics have been found in multiple media such as in oceans, rivers, estuaries, lakes, the atmosphere, beaches, sea ice, and sediments^6–10^. These small plastics originate either as primary sources from terrestrial runoffs, littering, and industrial discharge of particulates in commercial products in which they occur or as secondary sources from the degradation of large plastics^11–14^ (macroplastics, i.e., >5 mm).

Microplastics affect both the environment and the organisms therein. Microplastics act as vectors for heavy metal contamination, and diseases, thus aggregating and increasing toxicity in the environment^15–17^. Aquatic biota such as plankton, fishes, crabs, clams, shrimps, and mussels ingest microplastics which clog their tissues and organs, thereby affecting their energy reserves, causing neurotoxicity, behavioral abnormalities, stunted growth, decrease reproductivity, and eventual death^18,19^. These ingested microplastics can also bioaccumulate in humans through the consumption of seafood, eventually leading to inflammation, cell damage, and oxidative stress in humans^20,21^. Recently, there have been reported findings of microplastics in human placenta with dire effects on fetal development^22^. The breakdown of microplastics can result in the leaching of toxins which seeps into sediments or kill organisms^23,24^.

In addition to the harm to aquatic organisms and the environment, microplastics pollution affects economies in many ways, including clean-up costs, decline in fisheries and coastal tourism^25–27^. Over time, lost fishing gear breaks down through abrasion and biofouling resulting in the release of microplastic fragments and fibers^24^. Fishes consuming these pieces of microplastics can expose themselves to toxic chemicals^28,29^. Seafood is the main source of animal protein for approximately 20% of the global population^30^ (1.4 billion people). Marine microplastics therefore endanger this source of protein by reducing the efficiency and productivity of aquaculture and commercial fisheries through fish mortality.

Borrelle *et al*.^31^ estimates that about 19 to 23 million metric tons, or 11%, of plastic waste (i.e., the main source of microplastics) generated globally in 2016, entered aquatic ecosystems, with this estimate expected to increase to 53 million metric tons per year by 2030. Beaumont *et al*.^30^ estimates a loss in marine ecosystem services between $3,300-$33,000 for each metric ton of plastic entering the ocean per year. At these rates, the economic cost of marine plastic pollution runs into several billions of dollars per year.

**Fig. 1:**
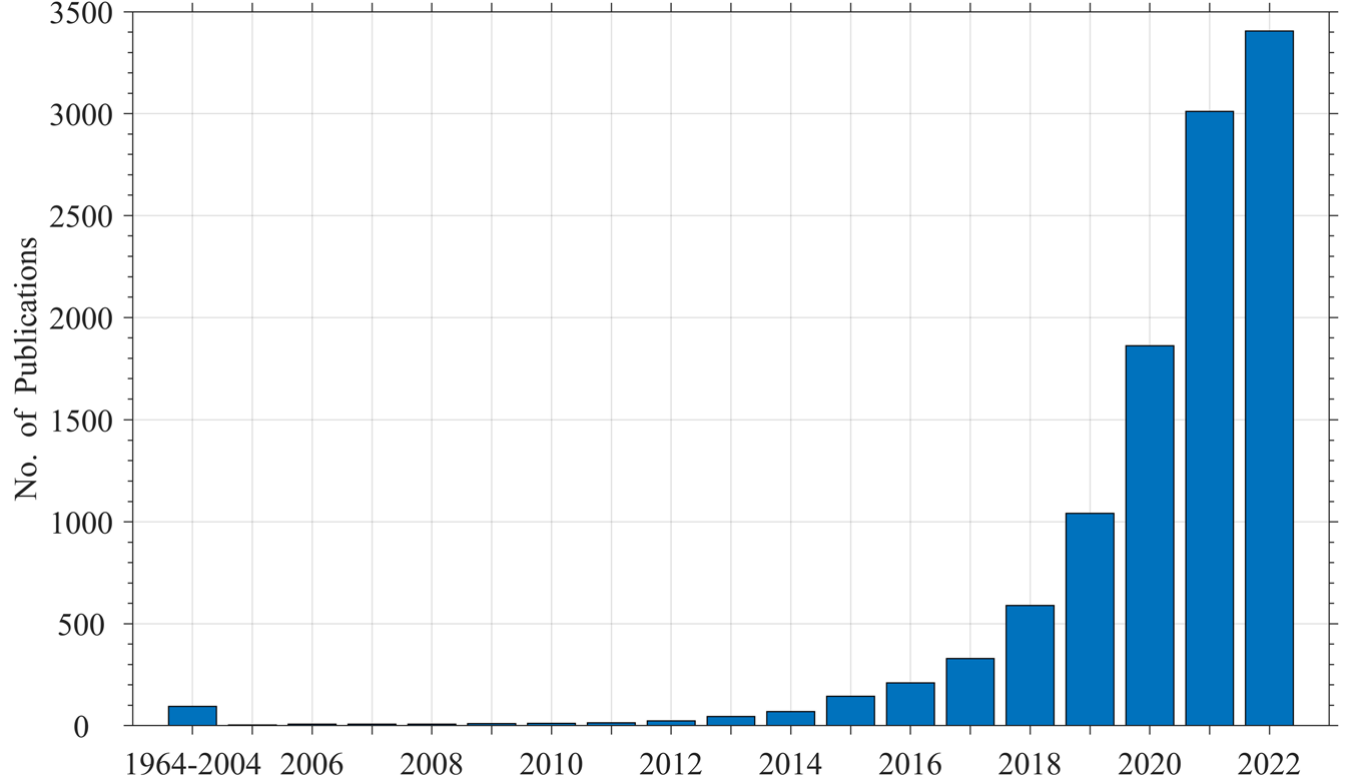
Number of microplastic publications between 1964–2022.

The increasing concern about microplastic pollution has led to a rapid research growth in this area in recent years, generating a large volume of data. To illustrate this trend, a Web of Science (WoS) database search using the keywords microplastic OR microplastics, along with the “All Fields” option was performed. Considering only English language “Articles” and “Review Articles” related to environmental microplastics, the search yielded 10,883 articles published between 1964 (first record of publication in WoS) and 2022 (Fig. 1). Among these articles, less than a hundred papers were published during the first four decades of the record keeping. Thereafter, the number of publications gradually increased until a rapid growth in the last five years. Indeed, the number of publications in 2022 (i.e., 3,405) was over three-fold that of 2019 (i.e., 1,042) (Fig. 1).

Despite the growing awareness and increase in microplastic research, a lack of large-scale, long-term, comprehensive data hinder a complete understanding of the sources, distribution, and impacts of microplastics. Even when available, the management of marine debris data, from large size visual surveys along the coast and in the open ocean, to effects of microplastics on planktonic communities, the blue economy, among others, lags far behind the needs of the scientific, education, and decision-maker communities^27,32^. The European Union’s EMODnet (European Marine Observation and Data Network) marine litter database^33^ (https://emodnet.ec.europa.eu/en/chemistry) archives and offers downloadable microplastic data as part of its floating microlitter collection. This database is however limited to only data from European waters. Another product, LITTERBASE^34^ (https://litterbase.awi.de/), developed by the Alfred Wegener Institute Helmholtz Centre for Polar and Marine Research, Germany, offers a global map and data analysis of marine microplastics from peer-reviewed scientific publications and a limited number of reports. This product does not however include non-published data, archives original data nor offers users the ability to download the data. A proposed ocean surface microplastic database by the Ministry of Environment of Japan (MOEJ) is also yet to be launched. The lack of comprehensive data on the spatial and temporal variability of microplastics is also a challenge for numerical modeling of their occurrence as a way to effectively understand and forecast their origins, trajectory, and aggregation^23,35^. Subsequently, there is the need for a well curated, expansive, and FAIR^36^ (Findable, Accessible, Interoperable, and Reusable) database to facilitate the understanding and control of microplastic pollution.

The National Oceanic and Atmospheric Administration (NOAA) National Centers for Environmental Information (NCEI)’s microplastics data stewardship project was started in January 2020 to obtain, aggregate, and archive global microplastic data. The microplastics website and database were launched in July 2021. This database collates microplastic data from large ocean surveys, citizen-science led initiatives, and published literature sources, which provides students, scientists, environmentalists, policy makers, and others, a robust, and open access repository for archived information needed in marine microplastics debris monitoring. One priority in creating the NOAA NCEI microplastic database is data access. The increased awareness of microplastic impacts on the environment and human health has led to a surge in microplastic research. Therefore, open access to the large amount of data generated is crucial to enable a broad, comprehensive assessment of the environmental issue. A FAIR microplastic database will enhance a uniform global understanding of the environmental problem^36–39^. In turn, it will aid in formulating management policies around the generation, handling, and disposal of microplastics.

A recent study by Jenkins *et al*.^39^ reported that only 28.5% of microplastic publications since 2006 contained a data sharing statement. Of this number, 38.8% provided their study data in the paper’s supplementary material and 13.8% through a data repository. In summary, the need to improve open access to microplastic data is monumental. An overarching goal of the microplastics product is to establish NCEI as the primary location for open access, comprehensive, quality-controlled global microplastics data and information. This effort along with other NCEI archived data (e.g., Global Ocean Current Database, Blended Seawinds, World Ocean Database, etc.), will serve a diverse international customer base to attain a holistic understanding of the global microplastic problem. In this paper, we present the NOAA NCEI global marine microplastics database, its creation, quality control procedures, and future directions.

## Results

### Overview

The NOAA NCEI microplastics database contains only *in-situ* measured marine microplastic concentrations. Data from animal tissues, model output and laboratory experiments are not included. At present, the database contains data from only the surface ocean. Recognizing microplastics are not only in surface ocean waters, our future goal is to broaden the database to include data from different ocean depths, ocean sediments, and beaches. This expansion will enable a more comprehensive understanding of microplastics in the marine environment.

**Fig. 2:**
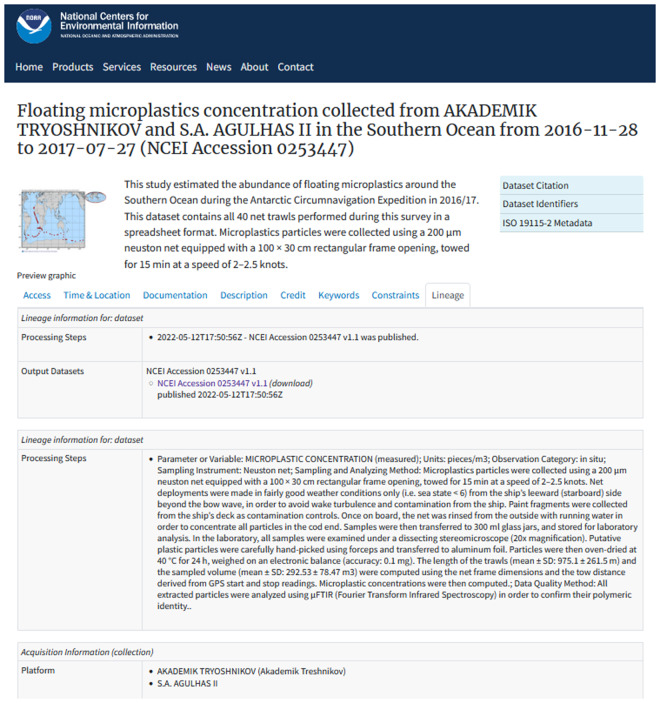
An example of a screenshot from an archived dataset collected in the Southern Ocean from 2016-11-28 to 2017-07-27, showing detailed information on how the data was collected, quality-controlled and analyzed. (Credit^9^: https://www.ncei.noaa.gov/archive/accession/0253447).

The database has two levels: archive and geodatabase. All microplastic data received are ingested into the NOAA NCEI archive after initial quality control and guaranteed to be available for at least 75 years. Next, the data are homogenized and added to the geodatabase which is displayed on the NCEI microplastics ArcGIS web portal. The archive provides more detailed information about individual datasets (Fig. 2), allowing in-depth exploration for interested categories of users such as scientists, graduate students, coastal managers, and policy makers. The ArcGIS geodatabase and web portal on the other hand is geared more towards a general audience. As such, not all metadata associated with an archived microplastic dataset is provided on the web portal.

### Archive display interface

A user-friendly interface displays the detailed metadata information about individual datasets in the archive. These information include a title for the data submission, investigators and their affiliations, package description, a map showing study area and sampling locations, data citation, temporal coverage, spatial coverage, platforms, keywords, identification information, funding information, and variable metadata section^40,41^. The variable metadata section contains details on how the data was collected, quality- controlled and analyzed (Fig. 2). The archive display interface also contains HTTPS and FTP links to download the data package.

To ensure uniformity and ease of use, the titles of archived datasets follow the following template: “[observed properties] collected from [research vessels or other platforms] in [sea names] from [start date] to [end date]. In the screenshot of an archive display interface shown in Fig. 2, the data package title is “*Floating microplastics concentration collected from AKADEMIK TRYOSHNIKOV and S.A. AGULHAS II in the Southern Ocean from 2016-11-28 to 2017-07-27*”^9^.

### ArcGIS geodatabase and web portal

The web portal contains the homogenized microplastic data. This interface uses user-friendly features such as dropdown menus, display filters, selection and drawing tools, and maps, to enhance the user experience of searching for microplastic data. A detailed help document is provided on the web portal to help users to navigate the site and download data.

As of June 2023, the database contains about 14,000 microplastic records. Each data record represents the concentration of microplastics (counts of pieces/m³) in a given space and time. Other information provided include the sampling equipment, collecting organization, key words associated with the record (e.g., ship name), and reference to original sources including bibliographic digital object identifiers (DOI) (Table 1). The database is publicly accessible from https://experience.arcgis.com/experience/b296879cc1984fda833a8acc93e31476 and can be downloaded (CSV, JSON, and GeoJSON formats) in its entirety or subsampled using filters (e.g. date, oceans, and seas, microplastic concentration, or sampling methods). The database is currently updated quarterly.

**Table 1:** Description of fields in the database and map portal.

Field	Description
Date	Numeric Month, Day, Year (e.g., 01/25/2023 for January 25, 2023) when the record was collected
Latitude	Geographical latitude in decimal degrees of the record location
Longitude	Geographical longitude in decimal degrees of the record location
Location-Oceans	Ocean in which the record occurred. Values are Arctic Ocean, Atlantic Ocean, Indian Ocean, Pacific Ocean, Southern Ocean
Location-Regions	Major Seas within an Ocean in which the record occurred. E.g., Arafura Sea; Bali Sea, Bay of Bengal, East China Sea, Gulf of Mexico, Mediterranean Sea, Tasman Sea
Location-SubRegions	Sub-regions of Ocean and Seas in which the record occurred. E.g., Adriatic Sea, Alboran Sea, Florida Keys National Marine Sanctuary, Strait of Gibraltar
Microplastics measurement	Concentration of the record
Unit	Concentration unit of the record: pieces/m^3^
Concentration class range	Concentration classification of the record
Concentration class text	Text description of the record concentration class range
Sampling Method	Instrument that was used to collect the record
Short Reference	Short form of reference for related publication for the record
Long Reference	Long form of reference for related publication for the record
DOI	Digital Object Identifier associated with related publication of the record
Organization	Institution that collected the record
Key words	Key search words associated with the record
NCEI Accession No.	Data package accession number associated with the record in NCEI archives
NCEI Accession No. Link	Link to the data package associated with the record in NCEI archives

Other details regarding data points may be accessed through the NCEI Accession No. Link.

The “NCEI Accession No. Link” directs the user to the original data package associated with the record in the NCEI archives. Here, the user can obtain in-depth information on how the record was obtained, quality controlled, and processed by the data collector.

With the “Concentration class range” and “Concentration class text”, we classify the microplastic concentrations (pieces/m³) in the database (Table 2). The classes are determined based on statistical characteristics and distributions of the database records such as minimum, mean, maximum, standard deviation, and interquartile range. The concentration class range and text of a record is therefore dynamic as more data is added and the statistical characteristics of the entire database change.

**Table 2:** Microplastic concentration class ranges and texts.

Concentration class range (pieces/m³)	Concentration class text
0-0.0005	Very low
0.0005–0.005	Low
0.005–1	Medium
1–10	High
>10	Very high

### Data sources

While it continues to grow, at the time of this manuscript writing, the NOAA NCEI microplastic database has collated information from 33 datasets, all from peer-reviewed published papers of 23 unique lead authors. 30 of the datasets were obtained by email solicitations while 3 were self-reported. 4 of the 33 datasets were collected by citizen science initiatives; *The Ocean Race* (formerly known as *Volvo Ocean Race*), *Adventure Scientists*, *Surfing for Science*, and *Oceaneye Association*. Most of the data records were collected from local and regional studies. Although the *Ocean Race* dataset provides a near-global snapshot of floating microplastic distribution, it does not cover all ocean sub-basins^42^.

### Spatial and temporal coverage

**Fig. 3:**
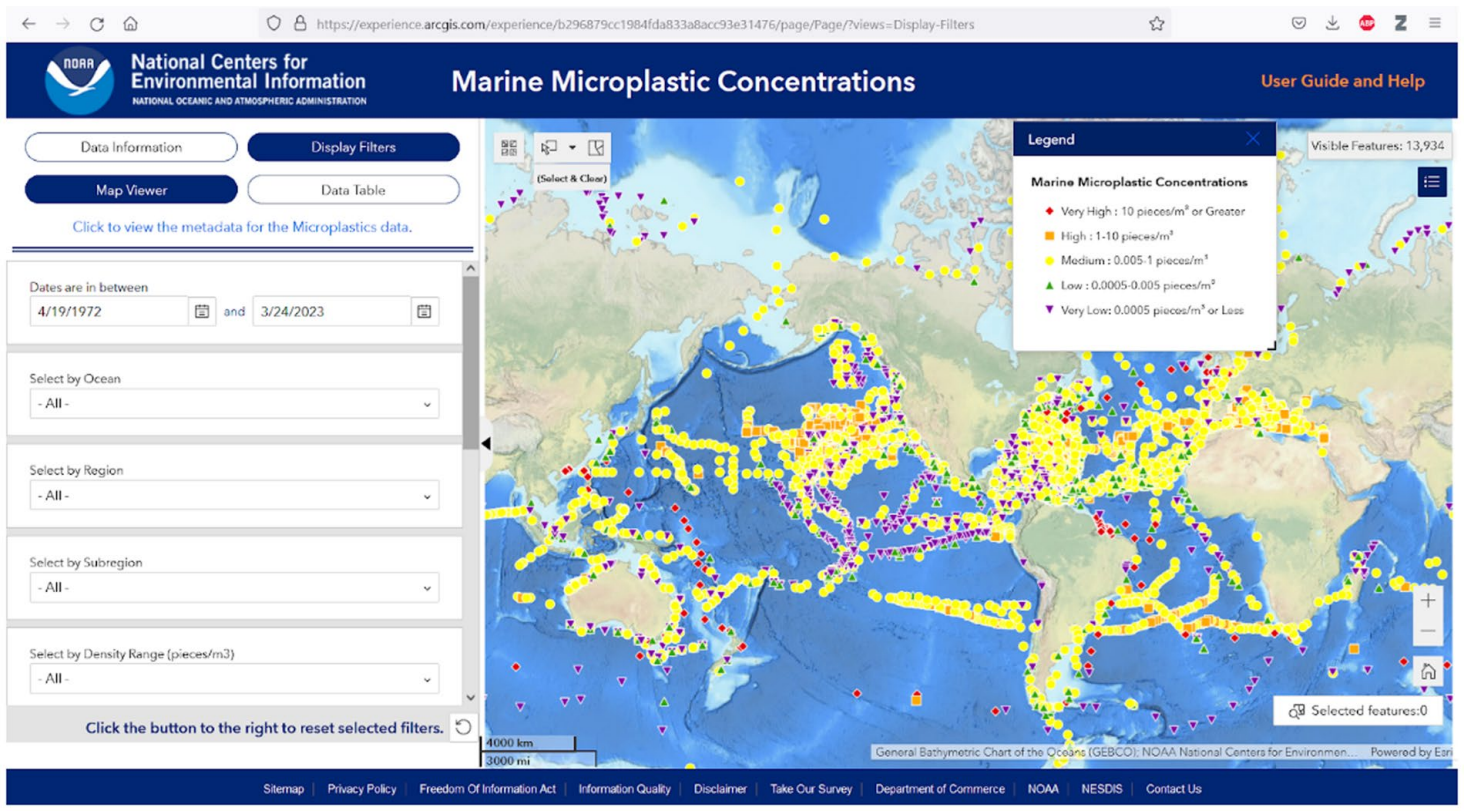
A screenshot showing the NOAA NCEI microplastic database GIS web portal with microplastic concentrations.

**Fig. 4:**
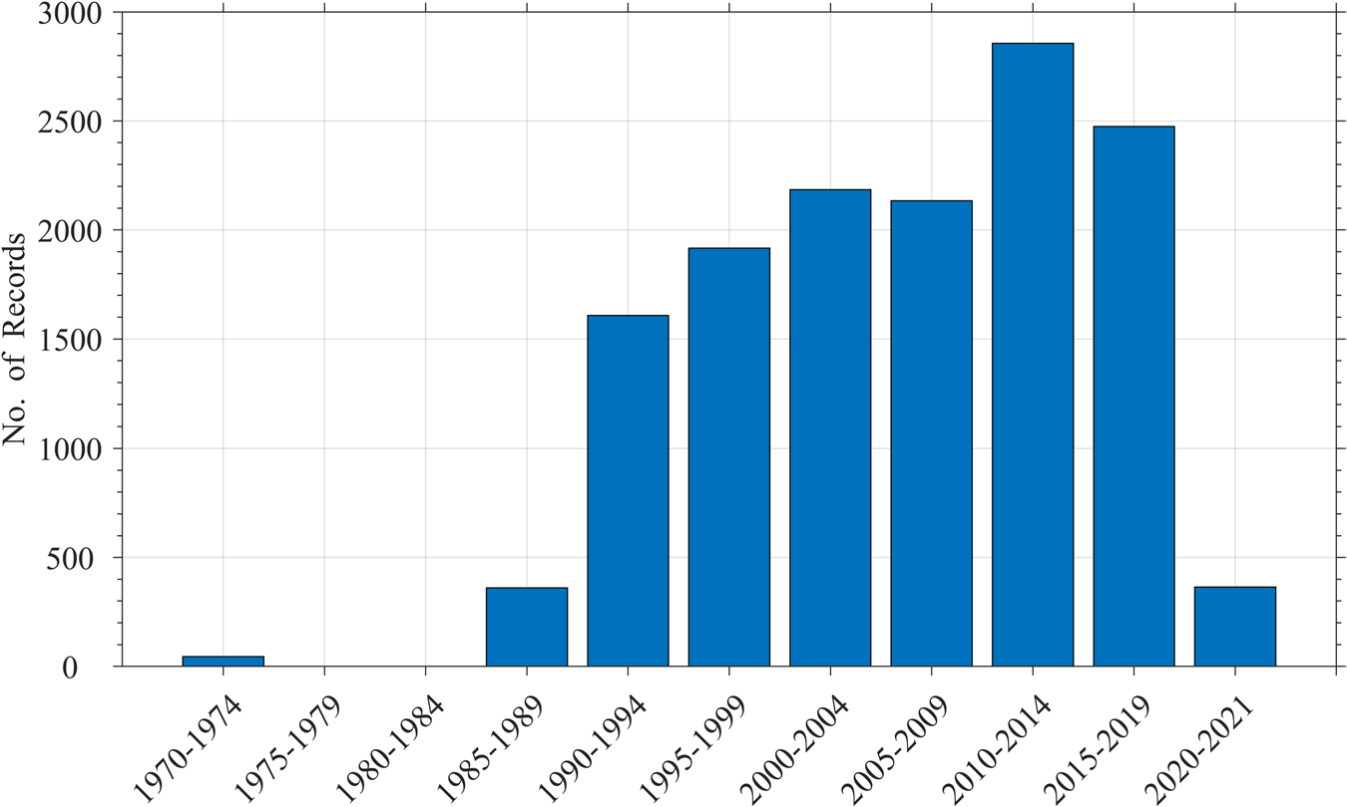
Number of microplastic records in the NOAA NCEI database.

The NOAA NCEI microplastic database is global, containing records from Arctic, Atlantic, Indian, Pacific, and Southern Oceans (Fig. 3). Most of the records are from the Atlantic Ocean (62%) with the least from the Southern Ocean (0.2%) (Table 3). At the time of this manuscript writing, the records were collected from 4/20/1972 to 10/5/2021, with the bulk (72%) collected in the post 2000 era (Fig. 4). Nearly all of the pre 2000 records were collected in the North Atlantic Ocean by the Sea Education Association (SEA), Massachusetts, USA^1^. The exceptions are ~45 records collected in the northeast Pacific Ocean in the 1970’s^43^.

**Table 3:** Number of microplastic records in each ocean.

Ocean	Microplastic Records
Atlantic	8,663
Pacific	4,784
Indian	325
Arctic	143
Southern	27
	**13,942**

## Discussion

As described in the Methods section, several steps are taken to ensure that the microplastic concentrations ingested into the NCEI database are of the highest standards. The NOAA NCEI *Send2NCEI*^44^ (S2N) data submission platform includes fields that allow only certain values and formats. This minimizes data entry and spelling errors. In addition, data submitters are contacted on ambiguities in their data such as duplicates, and outliers. Furthermore, the dataset is checked by multiple curators and subject matter experts, prior to being served to the public.

The field of microplastics research is quite young. Although there has been immense expansion of research activities and volume of data generated in recent times, there are still no uniform standards for data collection, analyses, and reporting. The growing interest in this contaminant has led to the development of several microplastic study methods, each with its own strengths and weaknesses^38^. Due to the stark variations in microplastic origin, density, chemical properties, morphology, size and color, there is no single combination of methods for sampling, extracting, analyzing, and reporting^38,39,45,46^. Thus, the microplastic concentrations in the database may not always be comparable across studies. Users should consider using data records along with more detailed metadata in the archives (such as sampling protocols and instrumental analysis, e.g., shown in Fig. 2) for further investigation of data usability.

### Importance of measuring and reporting standards

An example data compatibility issue observed while compiling the microplastics database is the inconsistency in data reporting standards such as the units of measurements. Units found in the literature include counts of pieces/m³, counts of pieces/km^2^, counts of pieces/km³, counts of pieces/g, g/km^2^, g/m^3^, among others. This lack of consistency creates problems for the research community and interest groups trying to compare records and to form composite datasets. Data harmonization will help merge multiple studies and synthesize information for a better understanding and regulation of the global microplastic problem. NCEI’s efforts to help address these shortcomings include providing a comprehensive microplastic database that gives an overview of the sampling efforts and helps identify the areas to standardize data collection and reporting to enable data harmonization. Standardization will help resolve the calibration needs for datasets with different methodologies, which will expand sharing, scalability, and utility of microplastic data. It will also enhance the fidelity and reproducibility of research results and success at obtaining grant funding for further studies. To achieve these, the standards ought to be consensus based, consistent, and based on best scientific practices.

The need and urgency to standardize and harmonize microplastic data collection, analysis, and reporting have led to a number of national and regional initiatives. Aside the NOAA NCEI’s effort, there are also the European Union’s EUROqCHARM (EUROpean quality Controlled Harmonization Assuring Reproducible Monitoring and assessment of plastic pollution; https://www.euroqcharm.eu/en) project, and the MOEJ guidelines for harmonizing ocean surface microplastic monitoring methods project^32,47^. On a global scale, the Global Partnership on Plastic Pollution and Marine Litter (GPML; https://www.gpmarinelitter.org/), a multi-stakeholder partnership under the United Nations Environmental Program, is leading efforts at bringing together all the aforementioned groups and others, unto a common platform for cooperation and coordination to share ideas, knowledge, experiences, and resources towards harmonizing microplastic data

Harmonization of current microplastic data products (i.e., EMODnet, LITTERBASE, and NCEI) starts by leveraging the common variables in the individual databases. These include sampling date, latitude, longitude, and sampling methods. Microplastic abundance is however not reported in common units among the different databases. Thus, data harmonization will involve performing unit conversions, among others, in order to have variables with a limited set of measurement units. Both the EMODnet and NCEI products provide users with access to the original and harmonized data while the LITTERBASE product does not archive the original data. In the case of the NCEI product, the archived data retains its original unit reported by the data owner while the harmonized data in the geodatabase (i.e., web portal) are converted to a common format (i.e., pieces/m^3^) where possible. For the LITTERBASE product, data is typically provided in units of items/km², items/km, items/m³ and other dimensions are converted to these units where possible for comparison. In a situation where microplastic measurements were provided in several dimensions (e.g., count and weight), LITTERBASE uses a preferred unit of items/km^2^. Also, for datasets that LITTERBASE considered to be spatially extensive, these were aggregated to means for subareas^34^. In summary, a unified guideline is needed in order to provide a FAIR and homogenized global microplastic data.

### Citizen science vs professional scientific research studies

Most of the records we have were obtained from professional scientific research studies, which can be time consuming, expensive, challenging, geographically limited, and seasonally driven^48,49^. There is, however, a growing interest and potential from citizen science initiatives for microplastic data collection^48–52^. When properly trained and harnessed, the enthusiasm of these groups can generate substantial data which will contribute towards a more informed, comprehensive understanding of microplastic occurrence and distribution. Involving citizen scientists also creates awareness outside of the professional scientific research community, increases engagement on environmental issues and promotes a community-based approach to environmental pollution management^48,49^.

Citizen science initiatives often adopt innovative measures to involve individuals through social and sports activities to collect microplastic samples. For example, the *Surfing for Science* citizen science project attached affordable and easy to use manta trawls on paddle surf boards, kayaks, and rowing boats to acquire microplastic samples^3^. Similarly, *The Ocean Race* initiative used two yachts (*Turn the Tide on Plastic* and *AkzoNobel*) that were competing in a race around the world as ships of opportunity to collect 96 microplastic samples during their circumnavigation^42^. The *Adventure Scientists* initiative used trained citizen scientists for an opportunistic collection of 1,628 1-liter glass jar grab samples across several locations such as shorelines, estuaries and offshore^49^.

## Methods

### Data acquisition and submission

At a minimum, we require data with sampling dates (year, month, and day), sampling location geographic coordinates, mesh size, and microplastic concentrations. Data submission and inclusion in the NOAA NCEI microplastic database is freely opened to the public. It is not restricted to only US-based researchers, or projects funded by NOAA or other US funding agencies. Data generated from both grant funded, and non-grant funded projects are welcome. Likewise, data from professional or non-professional scientists (e.g., citizen scientists) are all welcome. Both published and unpublished microplastic datasets are accepted and included in the database. All of the above data sources and kinds are subjected to the same rigorous quality assessment and quality control standards.

We obtain microplastic data predominantly in two ways; self-reporting by data owners and email solicitation requests to data owners. Self-reporting is typically done through the NCEI S2N web portal. This is an archiving tool that allows the data owner to easily submit their data files, metadata, and related documentation to NCEI for long term preservation, stewardship, and access. S2N thus helps the data owner meet any funding requirements for data documentation, sharing, and archiving^44^. S2N includes controlled vocabularies that enables accurate data findability. It also allows the creation of a user profile which enhances data submitter’s ease of use by retaining records of previous submissions and allowing it to be duplicated to start a new submission.

Data acquisition through email requests begins by NCEI scientists identifying suitable microplastic datasets. The scientists perform literature searches from online reference and citation databases such as Web of Science, Scopus, and Google Scholar using the keywords microplastic, microplastics, plastic, and plastics in the title, abstract and keywords. Identified research papers are then reviewed to ensure they (1) contain microplastic data, (2) are collected from the ambient marine water environment, (3) do not include data from animal tissues, (4) are *in-situ* data and not model output or laboratory experiment, and (5) use appropriate sampling and analytical methodologies such as those outlined below. If a paper is suitable, the corresponding authors are contacted through emails to obtain their permissions for data to be included into the NOAA archive and geodatabase, and freely and openly redistributed without restriction. When permission is granted, the data are archived on behalf of the owner using S2N. If an identified, suitable research paper uses secondary data, we contact the original data owner for their permission and cite the original data owner.

In addition, we find unpublished data by making inquiries to specific Citizen science groups, initiatives, and researchers. This includes direct contact with presenters at webinars, workshops, and conferences. Those data sampling methods are reviewed against sampling protocols found in published literature. If the sampling methods and protocols are in line with those of peer review publications, they are ingested into both the archive and the geodatabase. If the methods used by a study are too different from what is widely adopted in the literature, the data is archived but not added to the geodatabase.

### Data licensing

The NOAA NCEI microplastics database publishes only data that the owners have given explicit permissions to be made completely open and freely available to the public. All submitted data are under conditions of Creative Commons (CC) CC0 (i.e., open access) and CC-BY 4.0 (i.e., cite data source) licenses, or their equivalents, wherein the data is completely open, freely accessible to the public, and users are asked to cite the original data source. Any license assigned by the data source is identified in the metadata maintained and redistributed by NCEI. NCEI does not assign data licenses of any type to original data acquired by NCEI because only the data source can provide the license for the original data, not NCEI. NCEI may transform, reconfigure, or otherwise do quality checks/flags on original source data prior to including that source data into the microplastics database, thus adding value to the overall quality of output data from the microplastics database. NCEI applies a CC0 license to the NCEI microplastics database product, which provides specific attribution for each data package that was contributed to develop the NCEI microplastics product. Because NCEI does not include original data in the NCEI data product that applies a more restrictive license, there is little likelihood of a conflict between an originator’s license and the NCEI license.

There are instances where some scientific journals require researchers to submit their data to a repository prior to submitting their manuscripts. In this case, NCEI can archive the data and not make it discoverable to the public. After the publication of the said manuscript, the author informs NCEI, and the data then becomes discoverable and freely available to the public.

Each dataset archived at NCEI has an associated data citation. In both the archives and microplastic web portal, citation is given to the data owner. The data citation is consistent with the guidelines and recommendations of FORCE11^53^ and DataCite (https://datacite.org/), and contains information such as list of authors, title of the data package, publication year, data repository, NCEI accession number, and an optional DOI^40,41^. For a submitted data that already has a DOI, that DOI is maintained. While DOI is highly recommended for all submitted datasets, for those that do not have one, the data owner is given the option of whether a DOI should be minted for it or not.

### Quality assessment and quality control

#### Evaluating sampling and analytical procedures

Both self-reported and solicited data are subjected to quality assessment and quality control to ensure correctness and completeness before archiving. At present, there are no globally-defined uniform standards for microplastic data. As such, we assess the study that collected the data by evaluating the sampling methods and strategy, sample size, sample handling, processing and storage, laboratory preparations, negative and positive controls, sample treatment, and particle and polymer identification^38,46,54–57^.

We check that the sampling methods and strategies are clearly defined and reproducible. Known microplastics sampling methods include selective sampling, volume-reduced sampling, and bulk sampling^6,58^. In selective sampling, microplastics are directly extracted from samples by visual identification. In volume-reduced sampling the samples are filtered or sieved at the sampling location and only the targeted components are transported to the laboratory. In bulk sampling, the entire volume of the sample is taken and is considered the best method when the abundance of microplastic is small^6^. Examples of instruments used for microplastics sampling include manta net, neuston net, plankton net, bongo net, multiple opening–closing net, continuous plankton recorder, aluminum bucket, stainless steel bucket, glass bottles and jars, and water pump/intake through vessel system^2,4,38^. We confirm that the mesh size used for sampling and/ filtering was less than 5 mm in order to capture microplastics. The most commonly used net mesh sizes are 333–335 µm^59^.

The water volume that was sampled should be reported to aid the computation of microplastic concentration. Sufficient water volume should be sampled as microplastics are heterogeneously distributed^60^. We assess that the sample volume size is representative of the sampling objectives, methods (instruments), strategy, and location. For example, grab sampling collects more microplastic particles than trawl nets. Also, smaller mesh sizes retain more microplastics than larger mesh sizes^2,45,61^. In one instance, Barrows *et al*.^2^ observed that grab sampling collected over three orders of magnitude more microplastics per volume of water and smaller sizes than neuston net sampling. Ideally, the study should collect replicate samples providing a measure of variability in sample collection and a statistically robust analysis of data^62^. The number of replicates and how they were nested within samples should also be reported.

We evaluate the procedures that were used to handle, store, and process the microplastic samples to ensure that contamination from the field and the laboratory (air, water, and materials) were eliminated. We ensure that the study used non-plastic instruments for data collection and for laboratory analysis^6,37,46^. Between the moment a sample is collected and examination in the laboratory, the sample should be stored on ice or frozen^46,56^. Samples can also be preserved in a glass container with ethanol, formalin, or formaldehyde^56^. Materials that were used such as equipment, tools, clothing, and work surfaces ought to be free of microplastics contamination. This includes wearing cotton or non-synthetic clothes, and thoroughly washing materials and cleaning work surfaces with ultrapure water (e.g., Milli-Q water) and filtered solvents^6,63,64^. The study must also report the use of field and laboratory blanks to account for procedural contamination^46,65^. The reported microplastic concentration should account for the controls by deducting the baseline by microplastic count, shape, color, and polymer type^65^.

We assess if the study adopted procedures that enhance particle identification and counting. Sample treatment includes organic digestion, density separation, sieving and filtering^62,66,67^. Sieving is usually enough for particles >300 µm as the sizes are large enough to allow for adequate sorting. Organic digestion may be needed to dissolve organic matter in some samples especially for the detection of small microplastics (typically <300 µm^56^). Organic digestion methods may include the wet peroxide oxidation (WPO) method which uses aqueous 0.05 M Fe (II) solution and 30% H_2_O_2_ solution to digest organic materials^63^. Other studies may involve the use of 10% KOH solution as well as enzymatic digestion methods^68^. Once organic materials are removed from the sample, the authors should mention what instruments were used for visual identification and quantification of microplastics. The instrument detection limits should also be reported.

We note if the study reports the shapes and polymer types of microplastics encountered. While not currently a focus in our database, it may be in the future as this field evolves. Microplastic shapes include fiber, fragment, film, foam, and pellet^2,38,56^. Microplastic polymer types include polypropylene (PP), low density polyethylene (LDPE), high density polyethylene (HDPE), polystyrene (PS), polyamide (PA; nylon), polyethylene terephthalate (PET), and polyvinyl chloride (PVC)^46,66,69^. Researchers should report confirmation of microplastics using chemical characterization methods such as Raman and Fourier-transform infrared (FTIR) spectroscopy^6^. Particle counts with confidence intervals, detection limits for the count and for minimum particle size, polymer types and percentages (of different polymer types, of synthetic vs natural material), and particle sizes should also be reported^56^. It is noted that not all samples collected in a study can be confirmed using these technologies due to logistical constraints, costs, etc. Nevertheless, a reasonable subsample should be confirmed for microplastic polymer type. Hermsen *et al*.^56^ recommends that for pre-sorted particles less than 100, all particles should be analyzed. For particles more than 100, at least 50% should be identified with a minimum of 100 particles.

#### Evaluating sampled data

After examining the sampling and analytical procedures, we evaluate the microplastic data. We check that the data contains the minimum requirements: sampling dates (year, month, and day), sampling location geographic coordinates, mesh size, and microplastic concentrations. Environmental (e.g., wind conditions) or logistical factors that may affect the interpretation of results should also be reported^70,71^. We check that the value of each record item matches the data type and confer with the data submitter on any ambiguity. We also verify that the data are plastics less than 5 mm, collected from the ocean surface and within valid geographical limits (i.e., latitude is between 90°S and 90°N and longitude is between 180°W and 180°E decimal degrees). Finally, we flag duplicate data for further consultation with the data submitter.

Sampled microplastic concentrations depend on factors such as study objectives, study area, sampling time, sampling instruments, sampling strategies, and analytical methods^2,38,57,61^. We ensure that the reported microplastic concentrations are within a reasonable range with respect to findings in published literature. Outlier data points (e.g., higher than usual ranges seen in published literature) are flagged for further consultation with the data submitter. We accept microplastic data that are reported in concentration units (i.e., counts of pieces per unit volume). Particle counts (as opposed to total mass/weight) are more convenient to link with toxicity studies since it makes it easier to calculate concentrations of specific microplastic types^46,62^. Concentration units other than counts of pieces/m³ (e.g., counts of pieces/km^2^, counts of pieces/km³) are converted to pieces/m³ (using information from the study such as dimensions of sample collection instrument) for data harmonization. Submitted microplastic data that are reported as weight are archived but not displayed on the geodatabase map portal due to harmonization challenges with other data.

Conversion of units from surface area (e.g., counts of pieces/km^2^) to volume (i.e., counts of pieces/m³) for data harmonization potentially creates biases and also limits comparison with some datasets. Microplastic measurements per unit area appears to be the commonly used unit for data collected with nets (i.e., areal sampling, e.g., Lavender Law *et al*.^2^; Reisser *et al*.^11^; Eriksen *et al*.^13^) while measurements per unit volume appears to be the commonly used unit for data collected by other means such as buckets, bottles, and pumps (i.e., point/station/grab sampling, e.g., Osorio *et al*.^72^; Setiti *et al*.^73^). Because our database contains data collected with all these different instruments and sampling methods, we convert to a common unit of measurements per unit volume for harmonization in the geodatabase (web portal), while maintaining the original unit in the archive. It should be mentioned that there are several datasets (e.g., Goldstein *et al*.^43^; Faure *et al*.^50^; de Haan *et al*.^3^; Suaria *et al*.^9^) where data was collected with nets and the submitted data from the owner are reported in both measurements per unit area and measurements per unit volume (i.e., the unit conversions in this instance were not done by NCEI).

Microplastic data unit conversion comes with challenges. For example, the water volume sampled by nets could be misrepresented as the position of a net’s frame varies over water surface, especially in the presence of waves, thus the net (or even a volumeter), may not be entirely submerged in the water. There are advantages and disadvantages of each of the different microplastic sampling methods (as we have previously mentioned) and the microplastic research community is still deliberating on a possible unified unit of measure and standards of reporting. One of our aims in creating this database is to aggregate the different data types and allied information, which will hopefully generate enough information to help the research and end-user communities reach a consensus on standards. We have a notice on our website and help pages alerting users to use the geodatabase alongside the archive which contains the data in its original units submitted by the data owner.

## Data Availability

The NOAA NCEI microplastic concentrations data is publicly available at https://experience.arcgis.com/experience/b296879cc1984fda833a8acc93e31476, under the CC-BY 4.0 license. The NOAA NCEI web portal can be viewed at https://www.ncei.noaa.gov/products/microplastics. Here, the user can also find a detailed help document to navigate the site and download data. Microplastic data owners can also find information and links here to submit their data for archiving and inclusion into the database.

## References

[1] Lavender Law K (2010). Plastic Accumulation in the North Atlantic Subtropical Gyre. Science.

[2] Barrows APW, Neumann CA, Berger ML, Shaw SD (2017). Grab vs. neuston tow net: a microplastic sampling performance comparison and possible advances in the field. Anal. Methods.

[3] de Haan WP, Sanchez-Vidal A, Canals M (2019). Floating microplastics and aggregate formation in the Western Mediterranean Sea. Mar. Pollut. Bull..

[4] Hale RC, Seeley ME, La Guardia MJ, Mai L, Zeng EYA (2020). Global perspective on microplastics. J. Geophys. Res.: Oceans.

[5] Uhrin AV, Schellinger J (2011). Marine debris impacts to a tidal fringing-marsh in North Carolina. Mar. Pollut. Bull..

[6] Hidalgo-Ruz V, Gutow L, Thompson RC, Thiel M (2012). Microplastics in the marine environment: a review of the methods used for identification and quantification. Environ. Sci. Technol..

[7] Lavender Law K (2014). Distribution of Surface Plastic Debris in the Eastern Pacific Ocean from an 11-Year Data Set. Environ. Sci. Technol..

[8] Jambeck JR (2015). Plastic waste inputs from land into the ocean. Science.

[9] Suaria G (2020). Floating macro- and microplastics around the Southern Ocean: Results from the Antarctic Circumnavigation Expedition. Environ. Int..

[10] Uhrin AV, Hong S, Burgess HK, Lim S, Dettloff K (2022). Towards a North Pacific long-term monitoring program for ocean plastic pollution: A systematic review and recommendations for shorelines. Environ. Pollut..

[11] Reisser J (2013). Marine Plastic Pollution in Waters around Australia: Characteristics, Concentrations, and Pathways. PLoS ONE.

[12] Zhu J (2019). Microplastic pollution in the Maowei Sea, a typical mariculture bay of China. Sci. Total Environ..

[13] Eriksen M (2014). Plastic Pollution in the World’s Oceans: More than 5 trillion plastic pieces weighing over 250,000 tons afloat at sea. PLoS ONE.

[14] Egger M (2020). A spatially variable scarcity of floating microplastics in the eastern North Pacific Ocean. Environ. Res. Lett..

[15] Andrady AL (2011). Microplastics in the marine environment. Mar. Pollut. Bull..

[16] Cole M, Lindeque P, Halsband C, Galloway TS (2011). Microplastics as contaminants in the marine environment: a review. Mar. Pollut. Bull..

[17] Brennecke D, Duarte B, Paiva F, Caçador I, Canning-Clode J (2016). Microplastics as vector for heavy metal contamination from the marine environment. Estuar. Coast. Shelf Sci..

[18] Wright SL, Rowe D, Thompson RC, Galloway TS (2013). Microplastic ingestion decreases energy reserves in marine worms. Curr. Biol..

[19] Gray AD, Wertz H, Leads RR, Weinstein JE (2018). Microplastic in two South Carolina estuaries: occurrence, distribution, and composition. Mar. Pollut. Bull..

[20] van Cauwenberghe L, Devriese L, Galgani F, Robbens J, Janssen CR (2015). Microplastics in sediments: a review of techniques, occurrence, and effects. Mar. Environ. Res..

[21] Vethaak AD, Leslie HA (2016). Plastic Debris is a Human Health Issue. Environ. Sci. Technol..

[22] Ragusa A (2021). Plasticenta: First evidence of microplastics in human placenta. Environ. Int.

[23] van Sebille E, England MH, Froyland G (2012). Origin, dynamics, and evolution of ocean garbage patches from observed surface drifters. Environ. Res. Lett..

[24] Koelmans AA, Besseling E, Foekema EM (2014). Leaching of plastic additives to marine organisms. Environ. Pollut..

[25] Dowarah K, Devipriya SP (2019). Microplastic prevalence in the beaches of Puducherry, India and its correlation with fishing and tourism/recreational activities. Mar. Pollut. Bull..

[26] NASEM. Reckoning with the U.S. Role in Global Ocean Plastic Waste. https://nap.nationalacademies.org/catalog/26132/reckoning-with-the-us-role-in-global-ocean-plastic-waste (2022).

[27] Mofokeng, R. P. et al. The future of ocean plastics: designing diverse collaboration frameworks. *ICES J. Mar. Sci*., fsad055, 10.1093/icesjms/fsad055 (2023).

[28] Bellas J, Martínez-Armental J, Martínez-Cámara A, Besada V, Martínez-Gómez C (2016). Ingestion of microplastics by demersal fish from the Spanish Atlantic and Mediterranean coasts. Mar. Pollut. Bull..

[29] Leslie HA, Brandsma SH, van Velzen MJ, Vethaak AD (2017). Microplastics en route: Field measurements in the Dutch river delta and Amsterdam canals, wastewater treatment plants, North Sea sediments and biota. Environ. Int..

[30] Beaumont NJ (2019). Global ecological, social, and economic impacts of marine plastics. Mar. Pollut. Bull..

[31] Borrelle SB (2020). Predicted growth in plastic waste exceeds efforts to mitigate plastic pollution. Science.

[32] Isobe A (2021). A multilevel dataset of microplastic abundance in the world’s upper ocean and the Laurentian Great Lakes. Micropl.&Nanopl..

[33] Jack ME (2019). EMODnet marine litter data management at pan-European scale. Ocean Coast. Manag..

[34] Bergmann, M., Tekman, M. B. & Gutow, L. LITTERBASE: An online portal for marine litter and microplastics and their implications for marine life/ Baztan, J., Jorgensen, B., Pahl, S., Thompson, R. & Vanderlinden, J. (editors), In: Fate and Impact of Microplastics in Marine Ecosystems, MICRO 2016, Amsterdam, Elsevier, 2 p.. 10.1016/B978-0-12-812271-6.00104-6 (2017).

[35] Sherman P, van Sebille E (2016). Modeling marine surface microplastic transport to assess optimal removal locations. Environ. Res. Lett..

[36] Wilkinson MD (2016). The FAIR Guiding Principles for Scientific Data Management and Stewardship. Sci. Data.

[37] GESAMP. Guidelines for the monitoring and assessment of plastic litter in the ocean. http://www.gesamp.org/publications/guidelines-for-the-monitoring-and-assessment-of-plastic-litter-in-the-ocean (2019).

[38] Cowger W (2020). Reporting guidelines to increase the reproducibility and comparability of research on microplastics. Appl. Spectrosc..

[39] Jenkins T (2022). Current state of microplastic pollution research data: trends in availability and sources of open data. Front. Environ. Sci..

[40] Jiang L-Q, O’Connor SA, Arzayus KM, Parsons AR (2015). A metadata template for ocean acidification data. Earth Syst. Sci. Data.

[41] Jiang L-Q (2023). The Ocean Carbon and Acidification Data System. Sci. Data.

[42] Tanhua T, Gutekunst SB, Biastoch A (2020). A near-synoptic survey of ocean microplastic concentration along an around-the world sailing race. PLoS One.

[43] Goldstein MC, Titmus AJ, Ford M (2013). Scales of spatial heterogeneity of plastic marine debris in the northeast Pacific Ocean. PLoS ONE.

[44] Woodard, K. & Mize, J. End-to-end data management services for access and discovery of NOAA’s research data. *OCEANS 2016 MTS/IEEE Monterey*, Monterey, CA, USA, 10.1109/OCEANS.2016.7761423 (2016).

[45] Watkins L, Sullivan PJ, Walter MT (2021). What you net depends on if you grab: A meta-analysis of sampling method’s impact on measured aquatic microplastic concentration. Environ. Sci. Technol..

[46] ITRC (Interstate Technology & Regulatory Council). Microplastics Team Materials. Washington, D.C.: Interstate Technology & Regulatory Council, MP Team. https://mp-1.itrcweb.org (2023).

[47] Michida, Y. et al. *Guidelines for harmonizing ocean surface microplastic monitoring methods*. Ministry of the Environment Japan: Tokyo, Japan, https://www.env.go.jp/content/900453438.pdf (2019).

[48] Zettler ER (2017). Incorporating citizen science to study plastics in the environment. Anal. Methods.

[49] Barrows APW, Cathey SE, Petersen CW (2018). Marine environment microfiber contamination: global patterns and the diversity of microparticle origins. Environ. Pollut..

[50] Faure F (2015). An evaluation of surface micro- and mesoplastic pollution in pelagic ecosystems of the Western Mediterranean Sea. Environ. Sci. Pollut. Res..

[51] Hoellein TJ, Westhoven M, Lyandres O, Cross J (2015). Abundance, and environmental drivers of anthropogenic litter on 5 Lake Michigan beaches: a study facilitated by citizen science data collection. J. of Great Lake Res..

[52] McKinley DC (2016). Citizen science can improve conservation science, natural resource management, and environmental protection. Biol. Conserv..

[53] Puebla I, Lowenberg D, WG FRDPE (2021). Joint FORCE11 & COPE Research Data Publishing Ethics Working Group Recommendations..

[54] Klimisch HJ, Andreae M, Tillmann U (1997). A systematic approach for evaluating the quality of experimental toxicological and ecotoxicological data. Regul. Toxicol. Pharm..

[55] Kase R, Korkaric M, Werner I, Ågerstrand M (2016). Criteria for reporting and evaluating ecotoxicity data (CRED): comparison and perception of the Klimisch and CRED methods for evaluating reliability and relevance of ecotoxicity studies. Environ. Sci. Eur..

[56] Hermsen E, Mintenig SM, Besseling E, Koelmans AA (2018). Quality criteria for the analysis of microplastic in biota samples: A critical review. Environ. Sci. Technol..

[57] de Ruijter VN, Redondo-Hasselerharm PE, Gouin T, Koelmans AA (2020). Quality criteria for microplastic effect studies in the context of risk assessment: A critical review. Environ. Sci. Technol..

[58] Xiang S, Xie Y, Sun X, Du H, Wang J (2022). Identification, and quantification of microplastics in aquaculture environment. Front. Mar. Sci..

[59] Tirkey A, Upadhyay LSB (2021). Microplastics: an overview on separation, identification, and characterization of microplastics. Mar. Pollut. Bull..

[60] Koelmans AA, Redondo-Hasselerharm PE, Nor NHM, Kooi M (2020). Solving the nonalignment of methods and approaches used in microplastic research to consistently characterize risk. Environ. Sci. Technol..

[61] Tamminga M, Stoewer S-C, Fischer EK (2019). On the representativeness of pump water samples versus manta sampling in microplastic analysis. Environ. Pollut..

[62] Miller E (2021). Recommended best practices for collecting, analyzing, and reporting microplastics in environmental media: lessons learned from comprehensive monitoring of San Francisco Bay. J. Hazard. Mater..

[63] Masura, J., Baker J., Foster G. & Arthur C. *Laboratory Methods for Analysis of Microplastics in the marine Environment: Recommendations for Quantifying Synthetic Particles in Waters and Sediments*, https://repository.library.noaa.gov/view/noaa/10296 (2015).

[64] Zheng Y (2019). Distribution characteristics of microplastics in the seawater and sediment: A case study in Jiaozhou Bay, China. Sci. Total Environ..

[65] Wright SL (2021). Development of screening criteria for microplastic particles in air and atmospheric deposition: critical review and applicability towards assessing human exposure. Micropl.&Nanopl..

[66] Prata JC, da Costa JP, Duarte AC, Rocha-Santos TAP (2019). Methods for sampling and detection of microplastics in water and sediment: A critical review. TrAC, Trends Anal. Chem. TRAC.

[67] Eitzen L, Ruhl AS, Jekel M (2020). Particle size and pre-treatment effects on polystyrene microplastic settlement in water: Implications for environmental behavior and ecotoxicological tests. Water.

[68] Foekema EM (2013). Plastic in North Sea fish. Environ. Sci. Technol..

[69] Araujo CF, Nolasco MM, Ribeiro AMP, Ribeiro-Claro PJA (2018). Identification of microplastics using Raman spectroscopy: latest developments and prospects. Water Res..

[70] Kukulka T, Proskurowski G, Morét-Ferguson S, Meyer DW, Law KL (2012). The effect of wind mixing on the vertical distribution of buoyant plastic debris. Geophys. Res. Lett..

[71] Martí E, Martin C, Cózar A, Duarte CM (2017). Low abundance of plastic fragments in the surface waters of the Red Sea. Front. Mar. Sci..

[72] Osorio ED, Tanchuling MAN, Diola MBLD (2021). Microplastics Occurrence in Surface Waters and Sediments in Five River Mouths of Manila Bay. Front. Environ. Sci..

[73] Setiti S (2021). Seasonal variation of microplastics density in Algerian surface waters (South-Western Mediterranean Sea). Mediterr. Mar. Sci.

